# Determining anion-quadrupole interactions among protein, DNA, and ligand molecules

**DOI:** 10.1186/1471-2105-12-S7-A5

**Published:** 2011-08-05

**Authors:** Jason B Harris, David D Jenkins, Jonathan Reyles, Stephanie Rickett, Jordan M Utley, Elizabeth E Howell, Jerome Baudry, Robert J Hinde

**Affiliations:** 1 Graduate School of Genome Science and Technology, University of Tennessee, Knoxville, TN, 37996, USA; 2 Department of Electrical Engineering and Computer Science, University of Tennessee, Knoxville, TN, 37996, USA; 3 Department of Biochemistry, Cellular, and Molecular Biology, University of Tennessee, Knoxville, TN, 37996, USA; 4 Center for Molecular Biophysics, Oak Ridge National Laboratory, TN, 37831, USA; 5 Department of Chemistry, University of Tennessee, Knoxville, TN, 37996, USA

## Background

An extensive search through the Protein Databank (about 4500 nonredundant structures) was previously completed within our lab to analyze the energetic and geometric characteristics of an understudied molecular interaction known as an anion-quadrupole (AQ) interaction. Such an interaction occurs when the positively charged edge of an aromatic ring, resulting from a quadruple moment (i.e., a dual dipole moment), renders the aromatic molecule noncovalently bound to a nearby anionic molecule. The study considered a very limited scenario of molecules that can participate in AQ interactions, consisting of the phenyl group of a phenylalanine (phe) amino acid as the aromatic participant and the carboxylate group of an aspartate (asp) or glutamate (glu) amino acid as the anionic participant. The results revealed anion-quadrupole pairs to be prevalent within most of the protein structures. It was also observed that the interaction energy for AQ pairs was heavily dependent on the angle between the anion and plane of the aromatic ring, favoring a more planar interaction.

In light of these critical observations being made from such a limited scenario, only phe-glu and phe-asp pairs and in a reduced sample set of the PDB, we are now continuing this work of identifying AQ interactions using a greatly expanded strategy. We are following these four aims: 1. Optimizing the AQ-search program to run in a semi-parallel fashion and on a large cluster of processors in order to handle larger analyses, 2. Adding to our search additional anionic participants which will include non-protein structures such as DNA and small ligands, 3. Studying a subset of the AQ pairs with molecular dynamics simulations in buried and solvent exposed environments to observe non-static behavioral traits as well as the reproducibility of AQ interactions by force field parameters. 4. Building an online database for public access to our data and search program.

